# Serotonin, neural markers, and memory

**DOI:** 10.3389/fphar.2015.00143

**Published:** 2015-07-21

**Authors:** Alfredo Meneses

**Affiliations:** Departamento de Farmacobiología, Centro de Investigación y de Estudios Avanzados del Instituto Politécnico NacionalMexico City, Mexico

**Keywords:** memory, drugs, neural markers

## Abstract

Diverse neuropsychiatric disorders present dysfunctional memory and no effective treatment exits for them; likely as result of the absence of neural markers associated to memory. Neurotransmitter systems and signaling pathways have been implicated in memory and dysfunctional memory; however, their role is poorly understood. Hence, neural markers and cerebral functions and dysfunctions are revised. To our knowledge no previous systematic works have been published addressing these issues. The interactions among behavioral tasks, control groups and molecular changes and/or pharmacological effects are mentioned. Neurotransmitter receptors and signaling pathways, during normal and abnormally functioning memory with an emphasis on the behavioral aspects of memory are revised. With focus on serotonin, since as it is a well characterized neurotransmitter, with multiple pharmacological tools, and well characterized downstream signaling in mammals' species. 5-HT_1A_, 5-HT_4_, 5-HT_5_, 5-HT_6_, and 5-HT_7_ receptors as well as SERT (serotonin transporter) seem to be useful neural markers and/or therapeutic targets. Certainly, if the mentioned evidence is replicated, then the translatability from preclinical and clinical studies to neural changes might be confirmed. Hypothesis and theories might provide appropriate limits and perspectives of evidence.

## Introduction

It should noted that while, memory formation and forgetting are functions of the brain (e.g., Fioravanti and Di Cesare, 1992; Wagner and Davachi, 2001; Wixted, 2004; Mansuy, 2005; Hardt et al., 2013; Hupbach, 2013; Callaghan et al., 2014; Li et al., 2015a); in contrast, diverse neuropsychiatric disorders present dysfunctional memory (Meyer-Lindenberg et al., 2012; Millan et al., 2012, 2014). AD is popular brain alteration presenting memory deficits and dementia and the leading cause of dementia, and a major public health priority; but dysfunctional memory is observed in other age-related neurodegenerative disorders, schizophrenia, post-traumatic stress disorder, strokes, etc. (Millan et al., 2014; Hashimoto, 2015). Certainly, no effective treatment for dysfunctional memory exists (e.g., Millan et al., 2012, 2014; Sun et al., 2015); likely due to the absence of neural markers associated to memory. Hence, memory, amnesia, forgetting (e.g., Tellez et al., 2012b) and AD (e.g., McConathy and Sheline, 2015; Muenchhoff et al., 2015; also Scarr et al., 2015) as well as mild cognitive impairment (MCI) (Eshkoor et al., 2015) require neural markers.

Certainly, AD is a very complex neuropsychiatric disorder, where memory becomes progressively dysfunctional (e.g., Solodkin and van Hoesen, 1997; Rodríguez et al., 2012) resulting in amnesia and dementia. In contrast, forgetting is unintentional process characterized as a failure to remember information or a rather strategic function of the brain that helps to reduce interference in the processing or retrieval of relevant information (Ludowiq et al., 2010). Likewise, forgetting as a physiological phenomenon occurs all the time (see McGaugh, 2013; see also Davis, 2010; Berry et al., 2012; Hardt et al., 2013; Kaku et al., 2013; Li and Richardson, 2013; Papenberg et al., 2013). However, the pharmacological and neuroanatomical bases of forgetting or memory have been little explored and as diverse neuropsychiatric disorders present dysfunctional memory, we are aiming potential neural markers.

For instance, phrasing neural markers and brain functions in PubMed (May 7, 21 and 29 or June 2, 2015) yield 318 or 319 (including 50 review papers) publications. Hence, herein, aiming clues about mapping neural markers link to cerebral functions and dysfunctions. Mainly memory formation, dysfunctional memory, and as forgetting, which has been little explored respect to neural markers. In spite of promissory findings, to our knowledge, no previous systematic works have been published addressing these issues. It should be noted that of the revised papers, several are rich in backgrounds and perspectives.

Examples illustrating the interaction among behavioral tasks (Box 1), control groups and molecular changes and/or pharmacological effects are mentioned in the following lines. Importantly, behavioral parameters, drug-treatment and cognitive processes interact in mammals (see below) and invertebrate species (e.g., Chen et al., 2014). Particularly the role of serotonin in memory: interactions with neurotransmitters and downstream signaling might be useful (e.g., Seyedabadi et al., 2014; Eskenazi et al., 2015). Although the focus herein are adult mammal animals; notwithstanding, important recent advances in invertebrate species, include Monje et al. (2013) reporting that flotillin-1 is an evolutionary-conserved memory-related protein up-regulated in implicit and explicit learning paradigms; thus, translational approach—from invertebrates to rodents—led to the identification of flotillin-1 as an evolutionary-conserved memory-related protein.

Box 1 **Factors responsible for inconsistencies among laboratories.**

Certainly, a number of factors might be produce similar results or be responsible for some inconsistencies among laboratories studying memory; which are complex and multi focal; which should provide an analytic framework offering key clues. Indeed, analysis of memory should include behavioral tasks, type of memory, the dynamic hierarchy of neural markers and brain areas involved in memory formation (e.g., Euston et al., 2012; Eskenazi et al., 2015) vs. no training, amnesia, anti-amnesic effects or forgetting (e.g., see below). Likewise, the species and the nature of behavioral task (e.g., appetitively or aversively motivated), curves of behavioral acquisition (i.e., multi-trial or two trials task) or patterns of behavioral responses (progressive vs. all or none response), cognitive demand (easy or difficult task), timing of drug administration (pre-training, post-training or pretest) and kind of drug (e.g., agonist or antagonist), protocols of training and testing together with neurobiological markers (e.g., Duewer et al., 1995; Patton, 1995) accompanying mnemonic processes deserve attention. Among the behavioral memory tasks available (e.g., Peele and Vincent, 1989; Myhrer, 2003; Lynch, 2004); importantly, the implementation of new instruments for measuring memory in behavioral tasks assists in gaining deeper insight into learning and memory processes (e.g., Cook et al., 2004; Walker et al., 2011; Markou et al., 2013; Leger et al., 2014; Wolf et al., 2014).

Actually, serotonin has pharmacological tools and well characterized downstream signaling in mammals' species (e.g., Marin et al., 2012; Borroto-Escuela et al., 2015; McCorvy and Roth, 2015); then serotonin and other neural markers are used for studying cerebral functions and dysfunctions (e.g., Tomie et al., 2003; Wellman et al., 2007; Cavallaro, 2008; Marcos et al., 2008; Da Silva Costa-Aze et al., 2012; Ménard and Quirion, 2012; Reichel et al., 2012; Rodríguez et al., 2012; Woods et al., 2012; Haahr et al., 2013; Alabdali et al., 2014; Freret et al., 2014; Kitamura et al., 2014; Kondo et al., 2014; Lecoutey et al., 2014; Leger et al., 2014; Seyedabadi et al., 2014; Leiser et al., 2015; Suzuki and Lucas, 2015; Westrich et al., 2015; Zilles et al., 2015). Evidence is organized according with 5-HT markers (i.e., receptors and transporter) but markers of other neurotransmission systems are included. Importantly, using well-established 5-HT neural markers (Blenau and Baumann, 2015; Lau et al., 2015; Müller and Homberg, 2015) might provide insights about known and novel markers and therapeutic targets. Müller and Homberg (2015) are providing an excellent analysis regarding 5-HT markers, drug use and addiction.

## Memory tasks and molecular changes

### Memory decline across aging

Ménard and Quirion (2012) using the Morris Water Maze (MWM) task, distinguish aged rats in two groups—memory-impaired (AI) and memory-unimpaired (AU) relative to 6-months old adult animals. Dysfunctional memory was associated to increased metabotropic glutamate receptors 5 (mGluR5) in hippocampal post-synaptic densities (PSD) (Table 1); Ménard and Quirion (2012) conclude that in successful cognitive aging (i.e., AU animals) present a critical role for mGluR5, Homer 1 proteins and downstream signaling pathways. Certainly, in terms of signaling respect to cognition-enhancing drug targets, insights are emerging (e.g., Seyedabadi et al., 2014; Gyurko et al., 2015; Ménard et al., 2015; Sun et al., 2015).

**Table 1:** **Memory task and molecular changes: unimpaired vs. impaired aging vs. adult rats**.

**Function/dysfunction**	**Major findings**	**References**
Following MWM training	Brain area: hippocampal (CA1)	Ménard and Quirion, 2012
**Groups**		
AI	-AI dysfunctional memory, ↑ in hippocampal (CA1) mGluR5 in PSD -Hippocampal up-regulated Homer 1a and 1b/c levels PSD	
AU	-AU had enhanced mGluR5 as well as Homer 1b/c stainings. - AU had higher PKCc, ERK, p70S6K, mTOR, and CREB activation. - AU higher expression of immediate early gene Arc/Arg3.1.	

*MWM, Morris Water Maze; Aging AU, memory unimpaired; aging impaired memory (IM), PSD, post-synaptic densities*.

### Autism: neuro-inflammation and neurotransmission impairment

Although, Alabdali et al. (2014) did find that serotonin or dopamine in platelet-free plasma not correlated with social and cognitive dysfunction. It should be noted that serotonin has multiple markers (see below). And, several neurochemical parameters might show sensitivity and specificity; thus contributing to earlier and more accurate diagnosis of dysfunctional memory in disease such autism, AD, and the identification of effective treatments (e.g., Sheline et al., 2014a,b; Strac et al., 2015).

### 5-HT systems

As already mentioned, serotonin (5-hydroxytryptamine, 5-HT) is one of the neurotransmitter well characterized in mammal species (e.g., Hoyer et al., 1994; Saulin et al., 2012; Borroto-Escuela et al., 2015; McCorvy and Roth, 2015), it has multiple neural markers, including receptors (i.e., 5-HT_1A/1B/1D_, 5-HT_2A/2B/2C_, 5-HT_3_, 5-HT_4_, 5-HT_5_, 5-HT_6_, and 5-HT_7_ receptors) and transporter (named SERT) as well as volume transmission. These 5-HT markers are present in brain areas involved in memory (e.g., Buhot et al., 2003a,b; Puig and Gulledge, 2011; Rodríguez et al., 2012; Barlow et al., 2015; Leiser et al., 2015), sentence compression (Zilles et al., 2015) and drug addiction (Müller and Homberg, 2015).

### Serotonergic gene regulation during learning and memory

In an elegant work, Cavallaro (2008) using DNA microarrays analyzed hippocampal 5-HT receptors in two behavioral memory tasks and different times (Table 2); observing differential expressions in 12 receptors (Htr1a, Htr1b, Htr1d, Htr1f, Htr2a, Htr2c, Htr3a, Htr4, Htr5a, Htr5b, Htr6, and Htr7). At least Htr2c, Htr3a and Htr6 receptors had significant changes relative to swimming control animals and water maze trained animals. Htr2c expression was reduced at 1 h and increased at 24 h following training. Htr3a-mRNA was increased at 24 h, whereas Htr6 was decreased at 6 h; as observed in autoshaping (see below). In passive avoidance task, three 5-HT receptors showed changes in expression respect to naive and trained animals (i.e., conditioned animals, CA). Indeed, the expression of Htr3a was increased, whereas those of Htr1b and Htr4 were decreased. Certainly, expression of 5-HT receptors were also observed in control groups subjected to physical activity and mild stress (naive vs. swimming controls in the water maze; naive vs. CSTA and USTA in passive avoidance); notwithstanding, memory consolidation produced different magnitudes (e.g., Htr2c in the water maze) often opposite trends than in control animals (e.g., Htr3a in both water maze and passive avoidance). Producing cumulative patterns of gene expression, associated to time and 5-HT subtype receptor (see Cavallaro, 2008). Importantly, apparently water maze memory requires slight 5-HT_7_ receptor expression within 1-h; and passive avoidance memory involves expression of 5-HT_1A−1F_, 5-HT_2A_, and 5-HT_5A_ receptors. Of course, remaining to determine if the suppression of the other 5-HT receptors is necessary. Certainly, the molecular requirements differ between water maze and passive avoidance.

**Table 2:** **5-HT_1A_ receptor**.

**Function/dysfunction**	**Findings:**			
**Memory tasks**	**Water maze**	**Passive avoidance**	**Fear conditioning^h^^,^^i^**	**Pavlovian autoshaping^a^**
			genetic variability within 5-HTR_1A_(rs6295)	Pavlovian/Instrumental autoshaping^b^^,^^d^
	memory impairment and variations in expression^e^	expression^c^	modulation of expression^i^	expression
recovery from dissociative amnesia	increase of 5-HT_1A_ receptor in cortical regions^g^			
object-location associations	lower right than left hippocampal binding potential is related to better memory performance^j^			
Morris water maze memory retrieval	expression^f^			

a *Tomie et al., 2003*;

b *Luna-Munguía et al., 2005*;

c *Cavallaro, 2008*;

d *Perez-Garcia and Meneses, 2009*;

e *Li et al., 2015b*;

f *Saroja et al., 2014*;

g *Kitamura et al., 2014*;

h *Baas and Heitland, 2014*;

i *Sase et al., 2015*;

j *Glikmann-Johnston et al., 2015*.

Notably, Zaldivar and Krichmar (2013) observe in behaviorally naïve (i.e., untrained) animals, neurotransmitters changing including 5-HT receptors expression in areas regarded to neuromodulation or memory (amygdala); revealing connectivity and receptor localization, and patterns of expression among neurotransmission systems, receptors and brain areas.

### 5-HT_1A_ receptor

Although 5-HT_1A_ receptor may serve as a biomarker for cognitive functioning and target for treatment of cognitive impairment; notwithstanding hitherto evidence remains sparse and inconsistent (Borg, 2008; Borg et al., 2009). Certainly, the situation is changing; e.g., Yoshimi et al. (2014) report that brexpiprazole, presents serotonin-dopamine activity, and 5-HT_1A_ receptor partial agonism, attenuates phencyclidine-induced cognitive deficits; an effect blocked by the selective 5-HT_1A_ receptor antagonist WAY-100,635 (which alone has no effect). Yoshimi et al. (2014) conclude that brexpiprazole could ameliorate cognitive deficits in schizophrenia and other neuropsychiatric diseases. Contrasting findings exist regarding the 5-HT_1A_ partial agonists (e.g., buspirone), which alone impair memory in normal subjects (Meneses, 1999) but some of them (e.g., tandospirone) might be useful in the treatment of schizophrenia pathophysiology (Sumiyoshi et al., 2008). And, as tandospirone (e.g., Baba et al., 2015) also has anti-amnesic effects or facilitate performance in difficult memory tasks; hence, 5-HT_1A_ partial agonists might be useful in the treatment of dysfunctional memory.

Certainly, while if 5-HT_1A_ receptor agonists, partial agonists, or antagonists might be used for memory alterations (e.g., Meneses and Perez-Garcia, 2007; Pittalà et al., 2015); functional selectivity or biased agonism is revealing important insights regarding 5-HT_1A_ and 5-HT_3A_ receptors (e.g., Vardy and Kenakin, 2014; McCorvy and Roth, 2015). For instance, van Goethem et al. (2015) study “biased,” 5-HT_1A_ receptor agonists in a novel object pattern separation task (relative to episodic memory); showing that by preferentially activating post-synaptic 5-HT_1A_ heteroreceptors, or raphe-nuclei autoreceptors are potential novel molecular targets for improving memory. Likewise, Stroth et al. (2015) report that arylpiperazine ligands of 5-HT_1A_ receptor preferentially affect cAMP signaling vs. β-arrestin-2 recruitment; proposing the development of signaling pathway-selective drugs targeting this receptor.

Notably, recovery from dissociative amnesia increases cortical 5-HT_1A_ receptor (Kitamura et al., 2014; Table 3). Likewise, memory in autoshaping task (see Box 2) also increases 5-HT_1A_ receptor expression in 14 brain areas, but decrements in 7 and no changes in 12 (Table 3); suggesting that upregulated, down-regulated, and “silence” 5-HT_1A_ receptor in brain areas form part of neural circuits engaged in memory formation; thus demonstrating a high degree of specificity and memory mapping.

**Table 3:** **5-HT_1B_ receptor**.

**Function/dysfunction**	**Findings**	**References**
**Following Groups**		
5-HT_1B_ receptor KO	Exhibit a task-dependent selective learning facilitation; indeed, selective facilitation/impairment depending on the cognitive demand and/or age-related decline in spatial learning (water maze) abilities	Buhot et al., 2003a,b; Wolff et al., 2003
Aggressive social model	High 5-HT1B receptor density in the BLA to predict high levels of aggression in observer rats	Suzuki and Lucas, 2015
Expression	Positive correlations in control subjects between creative ability and average 5-HT_1B_ receptor availability in gray matter	Varrone et al., 2015

*BLA, basolateral amygdala*.

Box 2 **Autoshaping tasks.**

Autoshaping memory tasks have been focus by several research groups (e.g., Brown and Jenkins, 1968; Myer and Hull, 1974; Atnip, 1977; Oscos et al., 1988; Bussey et al., 1997, 2013; Lindner et al., 2003; Vanover et al., 2004; Ballaz et al., 2007; Rodriguez et al., 2008; Walker and Foley, 2010; Walker et al., 2011; Tomie et al., 2012; Krynetskiy et al., 2013; Markou et al., 2013; Gallistel et al., 2014; Holland et al., 2014; Lesaint et al., 2014; Talpos et al., 2014; Eskenazi et al., 2015; Talpos and Shoaib, 2015; in several animal species (e.g., Wasserman, 1981) including humans (Wilcove and Miller, 1974; Pithers, 1985). According with Holland et al. (2014), “autoshaping” or “sign-tracking” phenomenon has recently attracted considerable attention as a platform for studying individual differences in impulsivity, drug sensitization, and other traits associated with vulnerability to drug addiction. Autoshaping has been also used for detecting effects induced by memory, amnesia, drugs, genetic variations, aging and neural markers (e.g., Tomie et al., 2003, 2012; Vanover et al., 2004; Rodriguez et al., 2008; Fitzpatrick et al., 2013; Markou et al., 2013; Talpos et al., 2014). Notably, autoshaping is an associative automatized learning task (see below), and during memory consolidation of Pavlovian/instrumental autoshaping learning task, dentate gyrus, hippocampal CA1, basolateral amygdaloid nucleus and prefrontal cortex are require (see below). It should be noted that an important innovation, and growingly popular method of assessing cognitive functions is the automated touchscreen platform (e.g., Abela et al., 2013; Talpos et al., 2014; Delotterie et al., 2015), used for diverse cognitive tasks, comparable those in employ in human subjects (Horner et al., 2013), including autoshaping (e.g., Gallistel et al., 2014; Talpos et al., 2014; Silverman et al., 2015).

Autoshaping learning tasks involve classical and instrumental conditioning (i.e., stimulus-stimulus and stimulus-responding conditioning). It should be mentioned that long-lasting memories are most efficiently formed by multiple training sessions separated by appropriately timed intervals. Autoshaping meets this criterion and it allows modeling of behavioral situations requiring integration of information obtained from sign- and goal-tracking settings; representing memory of self-taught settings (Meneses, 2013, 2014). Certainly, autoshaping tasks (Pavlovian or instrumental; and Pavlovian/instrumental may produce initial modest and/or variable levels of conditioned responses (CR). Importantly, memory formation, amnesia and forgetting in Pavlovian/instrumental paradigms are accompanied by changes in neural markers, including 5-HT, glutamate, dopamine, and GABA transporters expression levels (Tomie et al., 2003; Tellez et al., 2012a,b), 5-HT receptor expression and cAMP production (Meneses, 2013). Certainly, forgetting as therapeutic targets for dysfunctional memory it has been little explored. As above mentioned, similar results, including pharmacological and neurobiological changes to those reported in autoshaping have been described in other memory behavioral tasks (for review see King et al., 2008; Marcos et al., 2008; Da Silva Costa-Aze et al., 2012; Reichel et al., 2012; Woods et al., 2012; Haahr et al., 2013; Freret et al., 2014; Nasehi et al., 2014b; Seyedabadi et al., 2014; Subramaniyan et al., 2014; Wilkinson et al., 2014; Delotterie et al., 2015; Sase et al., 2015; Westrich et al., 2015).

**Behavioral parameters during STM and LTM**

In addition to measuring CR in autoshaping, head-pokes (HP) during each training/testing session and head-pokes during CS (HP-CS) have recorded. These parameters provide information about exploration activity (HP) and food- intake motivation (Tellez et al., 2012a). For instance, as CR becomes progressive, HP-CS provides information on the association of CS-US and CR-US.

**Maximum level of CR**

It should be noted that as animals present different levels of CR, these values are normalized and the maximal CR level attained for each rat at 48 h is considered as 100% of performance. This value is then used to calculate the proportion or percentage of CR observed at 1.5, 24, and 216 h and the data of 1.5 h and 24 h are used as illustration; and multiple comparisons, including memory, forgetting, time vs. treatments for all behavioral parameters (Meneses and Tellez, 2015).

**Memory, amnesia and forgetting and neural transporters analysis**

As already mentioned autoshaping procedures produce variable levels of CR and a number of laboratories have been using autoshaping. It should be noted that, reproducibility among studies is important and expected that to vary (e.g., Marcus, 2014).

Importantly, Glikmann-Johnston et al. (2015) report that hippocampal human asymmetry in 5-HT_1A_ receptor expression (using [^18^F] MPPF binding), accompanies memory for object-location associations; lower right than left hippocampal binding potential is related to better memory performance (Table 2). Aubert et al. (2013) also report that the dual 5-HT_1A/7_ receptor agonist 8-OH-DPAT increased transcription of adenylate cyclase 1 in the hippocampus (CA1), suggesting that memory function could play a role in altered pairmate interaction dynamics; and these changes might be caused by 8-OH-DPAT-induced up- or down-regulation of 5-HT_1A_ and 5-HT_7_ receptor in the medial prefrontal cortex and in the hippocampus (CA1), respectively; and according with Aubert et al. (2013); and such as hypothesis is supported by rodent studies that implicate 5-HT_7_ function in contextual learning and memory consolidation.

On the other hand, genetic variability within 5-HT_1A_ receptor (rs6295) is associated with contextual fear independent (Table 3) (Baas and Heitland, 2014). Likewise, Weber et al. (2015) report that conditional inactivation of the GLUA1-encoding Gria1 gene selectively in 5-HT neurons of adult mice (i.e., Gria1 5-HT-/- mice) exhibited a distinct anxiety phenotype but showed no alterations in locomotion, depression-like behavior, or learning and memory. Importantly, contextual fear task increases hippocampal AMPA-, GluN1- and 5-HT_1A−_ containing receptor complexes (Sase et al., 2015) (Table 3). In addition, Saroja et al. (2014) studied spatial memory retrieval and hippocampal monoamine receptor (MAR) complexes (including 5-HT_1A_ and 5-HT_7_ receptors, and dopamine D1 and D2 receptors and colocalizations) in mice of 3–12 and 18 months. D1, D2, and 5-HT_7_ containing receptor complex levels were decreasing with age while 5-HT_1A_ receptor-containing complex was increased. In addition, the time spent in the target quadrant (i.e., memory retrieval) correlated with D1, 5-HT_7_, and 5-HT_1A_ receptors complex expression. Saroja et al. (2014) conclude that individual monoamine receptors are linked to spatial memory retrieval and are modulated by age. This same group (Subramaniyan et al., 2015) reports that the receptor complex levels containing hippocampal GluN1 and GluN2A of NMDARs, GluA1 and GluA2 of AMPA receptors, nAch7 and the D1A dopamine receptors were elevated during spatial learning, whilst levels of GluA3 and 5-HT_1A_ receptor containing complexes were reduced. Thus, supporting that 5-HT_1A_ receptor is useful neurobiological marker of memory.

### Pavlovian autoshaping: 5-HT_1A_ and 5-HT_2_ receptors (binding sites)

Interestingly, Tomie et al. (2003), studied the effects of experience with Pavlovian autoshaping procedures (Box 2) on lever-press conditioned response (CR) performance and ^3^H-8-OH-DPAT-labeled binding of 5-HT_1A_ and probably 5-HT_7_ (it should be noted that this drug has affinity for 5-HT_7_, see below; Table 3); as well as ^125^I-LSD-labeled binding of 5-HT_2A_ receptors were evaluated in four groups of rats. The groups (Paired High CR and Paired Low CR) received Pavlovian autoshaping procedures wherein the presentation of a lever (conditioned stimulus, CS) was followed by the response-independent presentation of food (unconditioned stimulus, US). Group Paired High CR showed more rapid CR acquisition and higher asymptotic levels of lever-press autoshaping CR performance relative to Group Low CR. Group Omission received autoshaping with an omission contingency, such that performing the lever-press autoshaping CR resulted in the cancelation the food US, while Group Random received presentations of lever CS and food US randomly with respect to one another. Though Groups Omission and Random did not differ in lever-press autoshaping CR performance, Group Omission showed significantly lower levels of 5-HT_1A_ binding in post-synaptic areas (frontal cortex, septum, caudate putamen), as well as significantly higher plasma corticosterone levels than Group Random. In addition, Group Random showed higher levels of 5-HT_1A_ binding in pre-synaptic somatodendritic autoreceptors on dorsal raphe nucleus relative to the other three groups. Autoradiographic analysis of 5-HT_2A_ receptor binding revealed no significant differences between Groups Paired High CR and Paired Low CR or between Groups Omission and Random in any brain regions. Notably, although extensive Pavlovian autoshaping training (Tomie et al., 2003) failed to produce any correlation between 5-HT_1A_ or 5-HT_2A_ receptor expression and CR; however, regardless the number of CR, Tomie et al. (2003) demonstrated correlation between both receptors expression and paired CS–US presentations. These data are also indicating that the neuroanatomical, neurochemical, and behavioral basis of Pavlovian and Pavlovian/Instrumental Autoshaping (P/I-A) are different (see Box 2). Although the latter could be considered as an instance of system processing styles (i.e., S-S, S-R, and stimulus-reinforcer [S-Rf] learning; see White and McDonald, 2002); nevertheless, the association of CR and 5-HT markers (Tomie et al., 2003) is replicated (Pérez-García et al., 2006; Pérez-García and Meneses, 2008). Notably, similar associations are observed in the Morris Water Maze and passive avoidance tasks (Cavallaro, 2008). Hence, the evidence supports the notion that 5-HT_1A_ receptor provides diverse neurobiological markers, pharmacological and genetic tools that have been used to investigate a variety of functions and dysfunctions (for references Meneses and Liy-Salmeron, 2012). Likewise, 5-HT_1A_ receptor also is therapeutic target, it seems to be useful for detecting functional and dysfunctional memory, and co-expression with other neurotransmission systems and serotonergic receptors.

### 5-HT_1B/1D_ receptor

The Buhot et al. (2003a,b; Wolff et al., 2003) seminal work (see also Drago et al., 2010) showed that 5-HT_1B_ receptor knockout mice exhibit a task-dependent selective learning facilitation; depending on the cognitive demand and/or age-related decline of spatial learning abilities (Table 4). In addition, pharmacological evidence indicates a possible involvement of hippocampal CA1 5-HT_1B/1D_ and 5-HT_2A/2B/2C_ receptors in harmaline-induced amnesia (Nasehi et al., 2014a). And 5-HT_1B_ receptor activation disrupts delayed alternation (DAL) performance in mice (Woehrle et al., 2013) and chronic fluoxetine pretreatment blocks 5-HT_1B_ receptor- induced deficits; suggesting a 5-HT_1B_ receptor modulation in orbitofrontal-dependent DAL. The 5-HT_1B_-induced DAL deficits may provide a model for obsessive compulsive disorder (OCD; Woehrle et al., 2013). The above evidence is consistent with the possibility that 5-HT_1B_ receptor inverse agonists might be useful for reversing memory deficits (e.g., Meneses, 2001; Meneses and Tellez, 2015). Importantly, 5-HT_1B/1D_ receptor expression in the frontal cortex is correlated to memory impairment (Garcia-Alloza et al., 2004). Certainly, Drago et al. (2010) highlight that 5-HT_1B_ receptor is a candidate modulator of the mnemonic and motivationally related symptoms in psychiatric illnesses. Moreover, positive correlations exist between creative ability and 5-HT_1B_ receptor expression in gray matter of control subjects; as well as in Parkinson disease (PD) patients between depression and creative ability (Varrone et al., 2015); importantly, PD patients have poor semantic memory and creative ability (Varrone et al., 2015).

**Table 4:** **5HT_2A/2B/2C_ receptor**.

**Function/dysfunction**	**Findings**		**References**
Parkinson disease	MS-DB 5-HT_2A_ receptor activation enhanced WM, which may be due to changes in the activity of septohippocampal network and monoamine levels in the hippocampus and mPFC		Li et al., 2015a
Memory (match-to-sample task)	Cognition-induced modulation of serotonin in the OFC: PET study of 5-HT_2A_		Hautzel et al., 2011
Memory (Pavlovian autoshaping)	5-HT_2A_expression and CR(Pavlovian autoshaping) association		Tomie et al., 2003
Spatial-discrimination serial reversal learning	Individual variations of 5-HT_2A_ in the OFC and dorsal raphé nucleus		Barlow et al., 2015
Dopamine2 and 5-HT_2A_ receptor variants	DRD2 and HTR2A genetic variants together modulate physiological prefrontal efficiency during working memory and also modulate the response to antipsychotics		Blasi et al., 2015
Fmr1 KO mice (model of fragile X syndrome);	Combinations of 5-HT_2B_ or D1-Rs or 5HT_2A_ or D2-Rs (low doses)	Enhance Ras-PI3K/PKB signaling input, GluA1-dependent synaptic plasticity and learning in Fmr1 KO mice; without causing anxiety related side effects	Lim et al., 2014
5-HT_2B_ receptor expression	*Htr_2B_* ^−/−^ mice, as shown by deficits in sensorimotor gating, in selective attention, in social interactions and in learning and memory processes		Pitychoutis et al., 2015
Against epilepsy induced memory decline	Combined action at MT1/2 and 5HT_2C_ receptors, reduced the depolarization-evoked release of glutamate, strong neuroprotective action and possible antioxidant properties of agomelatine		Vimala et al., 2014
Chronic microwave-induced cognitive deficit	Variations of 5-HT_1A_ and 5-HT_2C_ receptors expressions		Li et al., 2015b

WM, working memory; OFC, orbitofrontal cortex; mPFC, medial prefrontal cortex; CR, conditioned responses; MT, melatonin.

### Neurobiological mechanisms in the observational learning of aggression

Suzuki and Lucas (2015) report that chronic passive exposure to aggression modifies expression of D2 receptor in the nucleus accumbens core (AcbC) and shell (AcbSh), and 5-HT_1B_ receptor in the medial (MeA), basomedial (BMA), and basolateral (BLA) amygdala. And increased aggressive behavior reduced D2 receptor in bilateral AcbSh. Likewise, regardless of exposure aggression length 5-HT_1B_ receptor was augmented in bilateral BLA. Finally, low D2 receptor expression in the AcbSh significantly interacted with high 5-HT_1B_ receptor density in the BLA, predicting high levels of aggression in observer animals (Table 4). Suzuki and Lucas (2015) conclude that the dopamine-serotonin or AcbSh-BLA interactions; may be risk factors for aggression in observers chronically witness aggressive interactions (Suzuki and Lucas, 2015). Clearly, 5-HT_1B_ receptor expression was useful in detecting learning and memory of aggression.

### 5-HT_2A/2B/2C_ receptors

Li et al. (2015a) report that 5-HT_2A_ receptor is highly expressed in the medial septum-diagonal band of Broca complex (MS-DB), especially in parvalbumin (PV)-positive neurons linked to hippocampal theta rhythm (involved in normal and dysfunctional memory of PD). The medial forebrain bundle (MFB) lesions impaired working memory, hippocampal theta, decreased firing rate and density of MS-DB PV-positive neurons, rhythm, and DA levels in septohippocampal system and medial prefrontal cortex (mPFC). Intra-MS-DB injection of the 5-HT_2A_ receptor agonist 4-Bromo-3,6-dimethoxybenzocyclobuten-1-yl) methylamine hydrobromide (TCB-2) enhanced working memory, producing the opposite effects in control and lesioned and shortening TCB-effects; implicating dysfunctional 5-HT_2A_ receptor. Li et al. (2015a) conclude that unilateral lesions of the MFB induced working memory deficit, and activation of MS-DB 5-HT_2A_ receptor enhanced working memory, and involve monoamine levels in the hippocampus and mPFC. In addition, in a controlled cross-over PET study using a delayed match-to-sample task and the 5-HT_2A_ receptor antagonist [^18^F] altanserin, Hautzel et al. (2011) report a cognition-induced modulation of serotonin in the orbitofrontal cortex (OFC). Importantly, Tomie et al. (2003) demonstrated an association between 5-HT_2A_ receptor expression and memory formation in Pavlovian autoshaping task. In addition, individual differences in impulsive action and 5-HT_2A_ receptor cortical variations have been noted (Fink et al., 2015). Also, D2 and 5-HT_2A_ receptors present genetic variants and modulate physiological prefrontal cortex efficiency during working memory and response to antipsychotics (Blasi et al., 2015). Moreover, although an association between 5-HT_2A_ receptor polymorphism (his452tyr) and memory performances in AD has been proposed; no differences in verbal memory were identified by Guglielmi et al. (2015).

Importantly, Barlow et al. (2015) report markers of serotonergic function in the orbitofrontal cortex and dorsal raphé nucleus predicting individual variation in spatial-discrimination serial reversal learning. These authors conclude that rats in the upper quintile of the distribution of perseverative responses during repeated S-R reversals have significantly reduced levels of the 5-HT metabolite, 5-hydroxy-indoleacetic acid, in the OFC. Additionally, 5-HT_2A_ receptor expression in the OFC of mid- and high-quintile rats was significantly reduced compared with rats in the low-quintile group. These perturbations were accompanied by an increase in the expression of monoamine oxidase-A (MAO-A) and MAO-B in the lateral OFC and by a decrease in the expression of MAO-A, MAO-B, and tryptophan hydroxylase in the dorsal raphé nucleus of highly perseverative rats. Barlow et al. (2015) found no evidence of significant differences in markers of DA and 5-HT function in the DMS or MAO expression in the ventral tegmental area of low- vs. high-perseverative rats; indicating that diminished serotonergic tone (probably, at least via 5-HT_2A_ receptor) in the OFC may be an endophenotype that predisposes to behavioral inflexibility and other forms of compulsive behavior (Barlow et al., 2015).

Moreover, Lim et al. (2014) investigated mechanisms of action of psychoactive drugs that modestly benefit the cognitive performance in fragile X patients (the most common form of inherited mental retardation); reporting that compounds activating 5HT_2B_ receptor (5HT_2B_) or dopamine (DA) subtype 1-like receptors (D1-Rs) and/or those inhibiting 5HT_2A_ or D2 receptors moderately enhance Ras-PI3K/PKB signaling input, GluA1-dependent synaptic plasticity, and learning in Fmr1 knockout mice (Lim et al., 2014). Unexpectedly, combinations of these 5-HT and DA compounds at low doses synergistically stimulate Ras-PI3K/PKB signal transduction and GluA1-dependent synaptic plasticity and remarkably restore normal learning in Fmr1 knockout mice without causing anxiety-related side effects. Lim et al. (2014) suggest that properly dosed and combined psychoactive drugs may effectively treat the cognitive impairment associated with fragile X syndrome. In addition, Htr2B^−/−^ mice show deficits in sensorimotor gating, selective attention, social interactions as well as in learning and memory (i.e., fear conditioning and novel object recognition: STM and LTM) (Pitychoutis et al., 2015).

Regarding 5-HT_2C_ receptor, Vimala et al. (2014) highlight that epilepsy affects negatively cognitive function, producing depression, anxiety, etc. Mentioning among other issues that agomelatine is a novel antidepressant acting as melatonin MT1 and MT2 receptor agonist and 5-HT_2C_ receptor antagonist; producing reduction in the depolarization-evoked release of glutamate, strong neuroprotective action and possible antioxidant effects (Vimala et al., 2014); producing hippocampal neuronal cell survival and neurogenesis, neuroprotective effect in hippocampus and frontal cortex and the antioxidant potential may contribute to the protective action of agomelatine against epilepsy induced memory decline (Vimala et al., 2014). In addition, Walker and Foley (2010) report that administration of the 5-HT_2C_ inverse agonist mianserin impaired autoshaped operant response on day 2 than any other agent tested. In addition, decreasing the length of the acquisition session to 1-h augmented the difficulty of the autoshaping task further modulating the consolidation effects produced by the 5-HT_2C_ ligands (Walker and Foley, 2010). Moreover, Li et al. (2015b) report that repeat exposition to 2.856 GHz microwaves (averaging 5–30 mW/cm^2^) affects spatial learning and memory function, morphology structure of the hippocampus, electroencephalogram (EEG) and neurotransmitter content (amino acid and monoamine); including expression of 5-HT_1A 2A, and 2C_ receptors. Li et al. (2015b) demonstrated that chronic exposure to microwave could induce dose-dependent deficit of spatial learning and memory and inhibition of brain electrical activity, the degeneration of hippocampus neurons, and the disturbance of neurotransmitters; including hippocampal and cortical expression of 5-HT_1A_ and 5-HT_2C_ receptors.

Importantly, 5-HT_2A/2B/2C_ receptors are useful detecting learning and memory changes and drug effects. Aloyo et al. (2009) remind us of inverse agonism at 5-HT_2A_ and 5-HT_2C_ receptors.

### 5-HT_3_ receptor

5-HT_3_ receptor antagonists (e.g., tropisetron, ondansetron) have a long dated antiamnesic effects, including attenuation of age-associated memory impairment (e.g., Costall and Naylor, 1992; see also Shimizu et al., 2013). Recent evidence, from preclinical studies suggests that the interaction between amyloid-β peptides (Aβ) and the α7 nicotinic acetylcholine receptor (α7 nAChR) (Hashimoto, 2015) (Table 5). And tropisetron is also a α7 nAChR agonist and 5-HT_3_ receptor antagonist; binding to amyloid precursor protein and enhancing memory in AD patients (Table 5). Importantly, 5-HT_3_ receptor antagonists have been useful in treatments such as chemotherapy-induced emesis to neuroprotection (Fakhfouri et al., 2014; Hashimoto, 2015). Certainly, subtypes of 5-HT_3_ receptor exist (Thompson, 2013); and their mechanisms are complex. For instance, Kozuska et al. (2014) deal with the multiple salt bridges in the intracellular domain of the 5HT_3A_ receptor and these interactions increase the overall rigidity of the receptor, stabilize its low conducting state and affect the ligand cooperativity; suggesting that the allosteric effects of these regions on the receptor may be involved in a possible “reverse” allosteric modulation of 5HT_3_ receptor. In addition, it should be noted that agonist- and antagonist-induced up-regulation of surface 5-HT_3A_ receptor (Morton et al., 2015).

**Table 5:** **5HT_3_ receptor antagonist, neuroprotection**.

**Function/dysfunction**	**Findings**	**References**
AD	Tropisetron, a potent α7 nAChR agonist and 5-HT_3_ receptor antagonist, also bound to the ectodomain of amyloid precursor protein. Furthermore, tropisetron promoted greater improvements in memory current AD therapeutic drugs AD.	Hashimoto, 2015
	In addition, tropisetron represents an attractive potential therapeutic drug to delay or prevent MCI and AD. This drug is also used for the treatment of chemotherapy-induced emesis	Fakhfouri et al., 2014
Aβ rat model of AD in MWM	- Tropisetron might have a neuroprotective effect; tropisetron attenuated Aβ-induced hippocampal neuroinflammation	Hashimoto, 2015
	Subtypes of 5-HT_3_ receptor	Thompson, 2013
KO 5-HT_3A_ receptor	Loss of exercise-induced hippocampal neurogenesis and antidepressant effects, but not of learning enhancement	Kondo et al., 2014

*AD, Alzheimer's disease; MCI, middle cognitive impairment; MWM, Morris water maze*.

Moreover, Kondo et al. (2014) studied 5-HT_3A_ receptor subunit-deficient (htr3a-/-) mice revealing loss of exercise-induced hippocampal neurogenesis and antidepressant effects, but not of learning enhancement (Table 5). Kondo et al. (2014) conclude that the 5-HT_3_ receptor is the critical target of 5-HT action in the brain following exercise, being indispensable for hippocampal neurogenesis and antidepressant effects induced by exercise.

### 5-HT_4_ receptor

It should be noted that earlier evidence indicated that 5-HT_4_ receptor decreased in AD (see Eglen et al., 1995). Activation of 5-HT_4_ receptor has pro-cognitive effects on memory tasks (e.g., Bockaert et al., 2011; Peñas-Cazorla and Vilaró, 2014; Ramirez et al., 2014; Claeysen et al., 2015). Notably, Madsen et al. (2011) observe cerebral 5-HT_4_ receptor up-regulation starts at a preclinical stage of dementia and it continues while dementia is still at a mild stage and these authors speculate that this upregulation may be a compensatory effect of decreased levels of interstitial 5-HT, increase acetylcholine release or to counteract Aβ accumulation and improved cognitive function. Hippocampal 5-HT4 receptor expression correlates inversely with human memory (Haarh et al., 2013; Table 6). Also, old rats have decreased 5-HT4 receptor expression and poor memory relative to adult (Table 6).

**Table 6:** **5HT_4_ receptor**.

**Function/dysfunction**	**Findings**	**References**
Memory	Activation has promnesic effects in rodents and humans	Haahr et al., 2013; Peñas-Cazorla and Vilaró, 2014
Mechanisms in cognition	Increased dendritic spines in the CA1 region of the hippocampus. Neuronal activity and increased release of acetylcholine in the prefrontal cortex and hippocampus. It is not synthesized in cholinergic cells Pre-training SL65.0155 enhances olfactory memory discrimination, inducing hippocampal growth dendritic spines; suggesting that selective 5-HT_4_ stimulation increases structural plasticity in learning activated hippocampal circuits	Restivo et al., 2008; Marchetti et al., 2011; Peñas-Cazorla and Vilaró, 2014
Changes with age	Old rats decreased 5-HT_4_ expression and poor memory relative to adult	Waeber et al., 1996; Manuel-Apolinar et al., 2005; Marchetti et al., 2011
Memory	Hippocampal 5-HT_4_ expression correlates inversely with memory in humans.	Haahr et al., 2013
AD	This receptor and β-amyloid protein are present in early stages of AD	Madsen et al., 2011

*AD, Alzheimer's disease*.

In addition, evidence suggests that serotonergic activity, via 5-HT4 receptors in hippocampal, striatum, and cortical areas, mediates memory function and provides further evidence for a complex and regionally specific regulation over 5-HT receptor expression during memory formation (Manuel-Apolinar et al., 2005).

Segu et al. (2010) report adaptive changes in cholinergic systems, which may circumvent the absence of 5-HT_4_ receptor to maintain long-term memory under baseline conditions. In contrast, despite of adaptive mechanisms, the absence of 5-HT_4_ receptor aggravates scopolamine-induced memory impairments. The mechanisms whereby 5-HT_4_ receptor mediates a tonic influence on ChAT activity and muscarinic receptors remain to be determined (Segu et al., 2010). Restivo et al. (2008) highlight that pharmacological modulation of synaptic efficacy is a prominent target in the identification of promnesic compounds and that pre-training administration of the 5-HT_4_ receptor partial agonist SL65.0155 enhances olfactory discrimination and potentiates learning-induced dendritic spine growth in the mouse hippocampus; without affecting spine density in the pseudo-trained mice and, by itself, it does not promote spine growth. Likewise, the 5-HT_4_ receptor antagonist RS39604 prior to SL65.0155 prevents both improved memory and additional formation of spines; thus confirming the 5-HT_4_ receptor specificity of the observed effects (Restivo et al., 2008); and these authors conclude that 5-HT_4_ receptor stimulation selectively increases experience-dependent structural plasticity in learning-activated hippocampal circuits.

Marchetti et al. (2011) have also highlighted that in developing rats as well as in rats ranging from 3 to 9 months of age, significant modifications of 5-HT_4_ receptor expression have been observed (for references see Marchetti et al., 2011). These same authors propone that the poor memory formation observed in aged rats (Marchetti et al., 2011). And corresponding decreases in 5-HT_4_ receptor expression in brain areas (e.g., hippocampus, amygdala, etc.) involved in memory formation, could explain improved memory, dendritic spines (Restivo et al., 2008), neuronal excitability and release of the neurotransmitter acetylcholine (Ach) (see Segu et al., 2010; Marchetti et al., 2011; Peñas-Cazorla and Vilaró, 2014). Clearly, 5-HT_4_ receptor is useful neural marker of dysfunctional and memory formation as well as therapeutic target. Moreover, studying 5-HT expression during memory formation is giving new fresh insights (e.g., Haahr et al., 2013). Importantly, Haahr et al. (2013) report that hippocampal 5-HT_4_ receptor expression correlates inversely with human memory performance.

### 5-HT_5_

As mentioned above, Cavallaro (2008) reported that passive avoidance memory involves expression of several 5-HT receptors, including 5-HT_5A_. 5-HT_5_ receptor occurs in brain areas implicated in learning and memory. Post-training administration of the 5-HT_5A_ receptor antagonist SB-6995516 decreased CR during short-term (STM; 1.5-h; at 0.1 mg/kg) and long-term memory (LTM; 24-h; at 3.0 mg/kg). Moreover, considering that there are no selective 5-HT_5A_ receptor agonists, next, diverse doses of the serotonin precursor l-tryptophan were studied during STM and LTM, showing that l-tryptophan (5–100 mg/kg) facilitated performance, particularly at 50 mg/kg. In interactions experiments, l-tryptophan (50 mg/kg) attenuated the impairment effect induced by SB-699551 (either 0.3 or 3.0 mg/kg) (Gonzalez et al., 2013). All together this evidence suggests that the blockade of 5-HT_5A_ receptor appear to be able to impair STM and LTM (24 h) in autoshaping task, while its stimulation might facilitate it. Of course further investigation is necessary, meanly with selective 5-HT_5A_ compounds (Gonzalez et al., 2013). Interestingly, Yamazaki et al. (2014, 2015) reported that a 5-HT_5A_ receptor antagonist ameliorates positive symptoms and cognitive impairment in animal models of schizophrenia and in aged rats and induced-amnesia. An analogous case is observed regarding 5-HT_1A_ partial agonists (see above).

Returning to 5-HT_5_ receptor, Karimi et al. (2013) report that it has long been known that hippocampal spatial memory and the ability to navigate through space are sexually dimorphic traits among mammals, and numerous studies have shown that these traits can be altered by means of sex hormone manipulation. Male and female rat pups were injected with estradiol and testosterone respectively, at early stage of their lives to examine the effect of sex hormone manipulation on mRNA expression of Slc9a4, Nr3c2, Htr5b, and Mas1; among other results, these authors report that expressions of these genes are strongly influenced by sex hormones in both the frontal cortex and hippocampus, especially in male hippocampus, in which expression of all genes were up-regulated. Htr5b was the gene that was affected only in the males (Karimi et al., 2013). Hence, considering the pharmacological evidence mentioned above, probably learning and memory might be affected in these animals.

### 5-HT_6_ receptor

Diverse 5-HT_6_ receptor antagonists produce promnesic and/or antiamnesic effects in conditions, such as memory formation, age-related cognitive impairments; memory deficits in models of diseases such as schizophrenia, PD and AD (e.g., King et al., 2008; Claeysen et al., 2015). However, not all papers report promnesic and/antiamnesic effects of 5-HT_6_ receptor antagonists (e.g., Thur et al., 2014) (Table 7); probably related to timing, drug and memory task used. Memory, aging, and AD modify 5-HT_6_ receptors and signaling cascades; and 5-HT_6_ drugs modulate memory, which is accompanied with neural changes. Indeed, in an elegant work Eskenazi et al. (2015) manipulated selectively overexpression of 5-HT_6_ receptor in either direct or indirect pathway striatal medium-spiny neurons (dMSN and iMSN, respectively), revealing that increased 5-HT_6_ receptor expression in iMSNs delays instrumental learning and in DLS facilitates behavioral flexibility after habitual responding. It should be noted that 5-HT_6_ receptor expression decreases during memory (e.g., Huerta-Rivas et al., 2010; Ramirez et al., 2014). In addition, de Bruin and Kruse (2015) suggest that cognition could be improved by 5-HT_6_ receptor antagonists, by increasing the number of NCAM PSA-immunoreactive neurons in the dendate gyrus, inhibit mTOR and Fyn-tyrosine kinase and interact with DARPP-32.

**Table 7:** **5-HT_6_ receptor**.

**Function/dysfunction**	**Findings**	**References**
Memory/models of diseases	Antagonism produce promnesic and/or antiamnesic effects, including memory formation, age-related cognitive impairments; memory deficits in models for diseases such as schizophrenia, Parkinson, and AD	Meneses et al., 2011a; Ramirez et al., 2014; but always see Thur et al., 2014
Memory, aging, and AD	Modify 5-HT_6_ receptor and signaling cascades	Ramirez et al., 2014
Expression	5-HT_6_ decreases during memory	Huerta-Rivas et al., 2010; Ramirez et al., 2014
Expression	Overall, increased 5-HT_6_ receptor expression in iMSNs slowed instrumental learning and in DLS facilitated behavioral flexibility after habitual responding	Eskenazi et al., 2015
Cognitive therapy	Idalopirdine antagonist administration improves memory in patients with moderate AD	Wilkinson et al., 2014; see also Ramirez et al., 2014
Mechanisms	Blocking this receptor decreases over-activation of mTOR when there are insults in early life rodent deficits associated this normalize the social and episodic memory	Dayer et al., 2015
Signaling molecules	Cdk5 activity regulated and controlled by this neuronal migration and neurite outgrowth. Cdk5 modulates the activity of Fyn, Jab1 and mTOR	Dayer et al., 2015
	SNX 14 is an endogenous negative regulator of 5-HT_6_ receptor, modulating its signaling and trafficking Also, SNX 14 internalizes and degrades 5-HT 6 receptor	Ha et al., 2015

*AD, Alzheimer's disease; Cdk5, cyclin-dependent kinase; mTOR, mammalian target of rapamycin; dMSN, direct or indirect, iMSM pathway medium-spiny neurons*.

Notably, 5-HT_6_ receptor antagonists are, among, serotonergic therapies for cognitive symptoms in AD (e.g., Ramirez et al., 2014). Indeed, Wilkinson et al. (2014) report safety and efficacy of idalopirdine, a 5-HT_6_ receptor antagonist, in patients with moderate AD. In addition, 5-HT_6_ receptor is providing new insights about plasticity (Dayer et al., 2015). For example, at early stages of neuronal development, expression of 5-HT_6_ receptor constitutively regulates the activity of the cyclin-dependent kinase (Cdk) 5 and, through this mechanism, controls cellular processes involved in circuit formation (e.g., neuronal migration, neurite outgrowth). In addition, 5-HT_6_ receptor modulates developmental targets, including Fyn, Jab1, and mammalian target of rapamycin (mTOR). In therapeutic terms such as blockade of pathological over-activation of the mTOR pathway induced by early life insults in rodents and normalizes the associated social and episodic memory deficits. It should be noted that 5-HT_6_ receptor and Cdk5; and the latter mediates neuronal differentiation (e.g., hippocampus, striatum) in an agonist-independent manner (Seo and Tsai, 2014). In addition, Ha et al. (2015) report that 5-HT_6_ receptor directly interacts with SNX14 (protein-coupled receptors/regulators of G protein signaling), which regulates internalization; degradation of 5-HT_6_ receptor and cAMP production. This finding might be related to the evidence that 5-HT_6_ receptor agonists and antagonists modulate cAMP production and improve memory formation (e.g., Meneses et al., 2011c). We do not know yet why 5-HT_6_ receptor agonists and antagonists (e.g., Woods et al., 2012) may facilitate memory or may reverse amnesia in some memory tasks. However, 5-HT_6_ receptor inverse agonist might be useful (e.g., Hostetler et al., 2014; but see also Benhamú et al., 2014).

### 5-HT_7_ receptor

Recently Nikiforuk (2015) is providing perspectives of 5-HT_7_ receptor in the search for treatments for CNS disorders: including normal and dysfunctional serotonin-induced phase shifting of the circadian rhythm control of memory as well as locomotor and exploratory activity, anxiety, depression; and Guseva et al. (2014) about molecular mechanisms responsible for the 5-HT_7_ receptor-mediated signaling. Gasbarri and Pompili (2014) noted that 5-HT_7_ receptor antagonism might have antiamnesic effects (see also Horisawa et al., 2013). Gasbarri et al. (2008) suggested that 5-HT_7_ receptor blockade had procognitive effect, when the learning task implicated a high degree of difficulty. Others report that 5-HT_7_ receptor agonists facilitate memory and have antiamnesic effects (Table 8); remaining clarifying why of the paradoxical effects.

**Table 8:** **5-HT_7_ receptor**.

**Function/dysfunction**	**Findings**	**References**
Brain development, autism, depression	Contributes to networks during development and in the mature brain remodel, thus participating in emotion and cognition	Ciranna and Catania, 2014; Guseva et al., 2014; Volpicelli et al., 2014; Nikiforuk, 2015
Memory/amnesia	Apparently 5-HT_7_ receptor agonists and antagonist might facilitate memory formation and/or have anti-amnesic effects	e.g., Nikiforuk, 2015
Amnesia	Antagonism might have antiamnesic effects	Tajiri et al., 2012; Waters et al., 2012; Horisawa et al., 2013; Nikiforuk et al., 2013; Gasbarri and Pompili, 2014; Westrich et al., 2015
Memory/amnesia	Agonism has procognitive and/or antiamnesic effects	Perez-García and Meneses, 2005; Pérez-García et al., 2006; Costa et al., 2012; Eriksson et al., 2012; Di Pilato et al., 2014; Freret et al., 2014; Ruocco et al., 2014; Meneses et al., 2015
Memory and mRNA expression	Higher level of expression of 5-HT_7_ receptor mRNAs in autoshaping-trained relative to untrained groups	Pérez-García et al., 2006
Memory time-course	Progressive memory and mRNA 5-HT_1A_ or 5-HT_7_ receptors expression monotonically augments or declines in prefrontal cortex, hippocampus and raphe nuclei, respectively	Perez-Garcia and Meneses, 2009
Aging and memory	Hypothesis: a decreased expression of 5-HT_7_ receptor in CA3 hippocampal could account for impairments of the shift between spatial strategies across aging	Beaudet et al., 2015
Signaling	Coupled to a G_*s*_ protein, stimulation activates the AC increased cAMP, in addition, 5-HT_7_ is associated to G12; a small GTPase protein of the Rho family. G_α*s*_ and G_α 12_ are involved in the regulation of TrkB expression by 5-HT_7_, depending on the model of study	Guseva et al., 2014; Samarajeewa et al., 2014
Monoamine complex and memory	D1, D2 and 5HT_7_ decreasing together with age, 5-HT_1A_ receptors containing complex MAR increase with age. The receptors MAR, 5-HT_7_, 5-HT_7A_and D1, correlate with changes in spatial memory, which are modulated by age	Saroja et al., 2014

*MWM, Morris Water Maze; MAR, monoamine receptor complex (i.e., D1, D2, and 5-HT_7_ containing receptors)*.

Notably, Saroja et al. (2014), highlight that although evidence about monoamine receptor (MAR) biochemistry and pharmacology in aging exists, work on MAR complexes rather than subunits is limited; in consequence, MAR complexes in hippocampi of three different age groups (3–12 and 18 months) in mice and to link MAR changes to spatial memory retrieval in the water maze were determine (Table 8). MAR complexes were separated in order to show the pattern of dopamine and 5-HT_1A_ and 5-HT_7_ receptors and colocalizations (Saroja et al., 2014). For instance, D1-D2 and 5-HT_7_ receptors containing receptor complex levels decreased with age while 5-HT_1A_ receptor-containing complex was increasing. D1, 5-HT_7_, and 5-HT_1A_ receptor complex correlated with good retrieval memory in the water maze; hence, individual monoamine receptors are linked to spatial memory and are modulated by age. However, Beaudet et al. (2015) mention that changes in the level of transcription of the 5-HT_7_ receptor mRNA did not account for the age-related difference observed at the protein level, at least in hippocampal CA3 region; besides, 5-HT_7_ receptor might also be putatively subjected, across aging, to modifications in their affinity or to changes in their coupling to G-proteins or other signaling pathways. Notably, Beaudet et al. (2015) suggest that **a** decreased expression of 5-HT_7_ receptor in CA3 hippocampal could account for impairments of the shift between spatial strategies across aging (Table 8).

Moreover, when the time-course (0–120 h) of autoshaped CR is progressive; then mRNA 5-HT_1A_ or 5-HT_7_ receptors expression is monotonically augmented or decreased in prefrontal cortex, hippocampus and raphe nuclei, respectively (Perez-Garcia and Meneses, 2009). Hence, 5-HT_1A_ and 5-HT_7_ receptors expression might be regulated by the level of memory formation and to be brain areas dependent. Moreover, the cyclic adenosine monophosphate (cAMP) is a second messenger and a central component of intracellular signaling pathways that regulate a wide range of biological functions, including memory (e.g., Kandel, 2001). And progressive time-course of memory formation in an autoshaping learning task (Pérez-García and Meneses, 2008); shows that *ex-vivo* cAMP production from trained and over-trained groups compared to untrained ones, the former group had the highest levels of cAMP and the latter rats showed increased production but less relative to trained rats. Importantly these changes varied according with normal memory or amnesia and brain areas; hence cAMP production is important in the signaling case in mammalian memory formation (Pérez-García and Meneses, 2008).

The above findings should be considered in the context that apparently 5-HT_7_ receptor agonists and antagonist (e.g., Nikiforuk, 2015) might facilitate memory formation and/or have anti-amnesic effects. Other interesting recent finding is that according with Rojas et al. (2014) serotonin regulates neurite outgrowth through 5-HT_1A_ and 5-HT_7_ receptors in cultured hippocampal neurons. Certainly, De Filippis et al. (2015) highlight that promnesic effects of the 5-HT_7_ receptor agonist LP-211 treatment strongly depend on the basal level of performance. Notably, Ruocco et al. (2014) report that 5-HT_7_ receptor stimulation improves selective spatial attention and produces permanent changes in several neural markers, including expression of glutamatergic receptors and dopamine transporter (DAT).

Very importantly, 5-HT7 receptor can form heterodimers with 5-HT1A receptors both *in-vitro* and *in-vivo* (see Guseva et al., 2014) and according with these authors, from the functional point of view, heterodimerization decreases Gi-protein coupling of 5-HT1A receptor and attenuates receptor-mediate deactivation of G-protein-gated potassium (GIRK) channels, without substantial changes in the coupling of 5-HT7 receptor to the Gs-protein. Moreover, heterodimerization significantly facilitated internalization of 5-HT1A receptor, while internalization kinetics of 5-HT7 receptor was decelerated upon heterodimerization (see Guseva et al., 2014).

## Factors responsible for inconsistencies among laboratories

### Neural transporters, memory, forgetting and drugs

**Figure 1:**
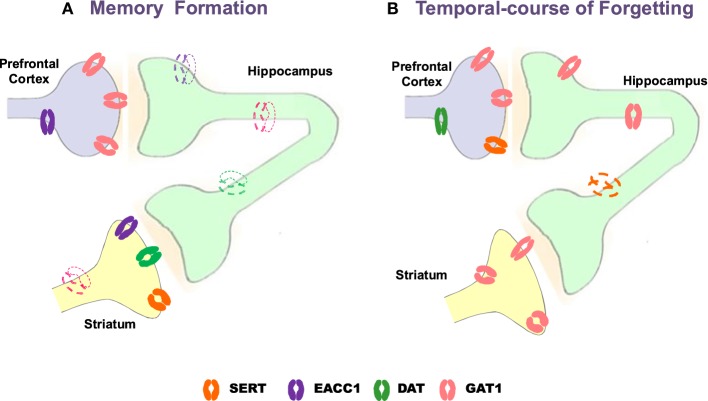
**Schematic representation of changes with Western blot analysis of neural transporters in prefrontal cortex, hippocampus and striatum during memory formation and temporal-course of forgetting**. Strong color refers to up-regulation, slight color refers to down-regulation. GAT1, GABA transporter 1; EAAC1, neuronal glutamate transporter excitatory amino acid carrier-1; DAT, dopamine transporter SERT, serotonin transporter (modified from Tellez et al., 2010, 2012a,b).

Notwithstanding neurotransmission systems are related to memory formation, amnesia and/or therapeutic targets for memory alterations, the role of transporters γ-aminobutyric acid (GABA, GAT1), glutamate (neuronal glutamate transporter excitatory amino acid carrier; EACC1), dopamine (DAT) and serotonin (SERT) is poorly understood. Emerging evidence indicates that memory formation (short- and long-term memory; STM and LTM, respectively) in a Pavlovian/instrumental autoshaping (see Box 1) is associated to up-regulation of prefrontal cortex GAT1 and EAAC1, striatal SERT, DAT and EACC1; while, hippocampal EACC1, GAT1, and SERT are down-regulated (Tellez et al., 2012a,b; Table 9; Figure 1). Moreover, pharmacological analysis shows that methamphetamine (METH)- induced amnesia down-regulated SERT, DAT, EACC1, and GAT1 in hippocampus and the GAT1 in striatum; no-changes are observed in prefrontal cortex. Fluoxetine (antidepressant, 5-HT uptake inhibitor) improved memory consolidation (particularly LTM), which is associated to DAT, GAT1 (prefrontal cortex) up-regulation, but GAT1 (striatum) and SERT (hippocampus) down-regulation. Fluoxetine plus METH prevented amnesia, which was associated to DAT, EACC1 and GAT1 (prefrontal cortex), SERT and DAT (hippocampus) and EACC1 or DAT (striatal) up-regulation.

**Table 9:** **Neural transporters during STM and LTM, amnesia (methamphetamine), forgetting, (fluoxetine) improved LTM, (fluoxetine) anti-forgetting effects and anti-amnesic (fluoxetine plus methamphetamine) effects**.

**Cognitive process**	**Neural transporters expression of transporters in brain areas**	**References Tellez et al., 2012a,b**
STM and LTM	Up-regulation of PFC GAT1 and EAAC1, striatal SERT, DAT and EACC1; while, HIP EACC1, GAT1 and SERT are down-regulated	
Amnesia	Down-regulated SERT, DAT, EACC1 and GAT1 in HIP the GAT1 in striatum; no-changes are observed in PFC	
Forgetting	Up-regulation of GAT1 (PFC and HIP) and DAT (PFC) while SERT (HIP) is down-regulated; no-changes are observed in striatum	
Improved LTM	DAT, GAT1 (PFC up-regulation), but GAT1 (striatum) and SERT (HIP) down-regulation	
Anti-forgetting effects	striatal GAT1 and HIP DAT up-regulation, but PFC GAT1 down-regulation	
Anti-amnesic effects	DAT, EACC1 and GAT1 (PFC), SERT and DAT (HIP) and EACC1 or DAT (striatal) up-regulation	

*STM, short-term memory; LTM, long-term memory; GAT1, GABA transporter 1; DAT, dopamine transporter; SERT, serotonin transporter; EACC1, glutamate transporter 1; PFC, prefrontal cortex; HIP, hippocampus*.

### Memory formation/forgetting and SERT expression

Forgetting in Pavlovian/instrumental autoshaping is associated to up-regulation of GAT1 (PFC and HIP) and DAT (PFC) while SERT (HIP) is down-regulated; no-changes are observed in striatum (Table 9). Methamphetamine alone not affected forgetting but up-regulates hippocampal DAT and EACC, prefrontal cortex DAT and striatal GAT1 or EACC1. Fluoxetine alone prevents forgetting, which is associated to striatal GAT1 and hippocampal DAT up-regulation, but prefrontal cortex GAT1 down-regulation. Fluoxetine plus METH prevent forgetting, which is associated to hippocampal DAT, prefrontal cortex SERT and striatal GAT1, DAT, or SERT up-regulation, but prefrontal cortex GAT1 down-regulation. Together these results show that forgetting provokes primarily hippocampal and prefrontal cortex transporters changes; it represents a cognitive process hardly modifiable and its prevention could causes different transporters expression patterns. Notably, together the results suggest that: (1) memory formation, amnesia and anti-amnesic effects are associated to specific patterns of transporters expression; (2) STM and LTM, forgetting and anti-forgetting effects are associated to specific patters of transporters expression and brain areas; (3) amnesia and forgetting affect different brain areas and produce differential patters of transporter expression. Hence, in pharmacological and neuroanatomical terms, amnesia and forgetting differ.

### Neural transporters and brain functions and dysfunctions

It should be noted that neural transporters regulate intra-synaptic levels of neurotransmitter, which allows a global picture of synapses. Moreover, diverse evidence indicates that memory formation, forgetting, amnesia, and/or anti-amnesic effects can also be modulated by changes in the expression of neurotransmitter transporters (e.g., Schmitt and Hiemke, 2002; Chen et al., 2011; Reichel et al., 2012; Yang et al., 2013). Hence, a brief overview of evidence involving GAT1, EAAT1, SERT, and DAT as well other neurobiological markers regarding memory and other cerebral functions is include.

### GAT 1

Attention deficit/hyperactivity disorder (ADHD) is featured by hyperactivity, impaired sustained attention, impulsivity, and usually varying degrees of dysfunctional learning and memory (see also Meneses et al., 2011b) and motor incoordination (Yang et al., 2013). Importantly, Yang et al. (2013) report that GAT1 gene knockout (KO) mouse (GAT1^−/−^) is hyperactive and exhibit impaired memory performance (Morris water maze). KO GAT1 mice have low levels of attentional focusing and increased impulsivity; the hyperactivity in these KO mice is reduced by both methylphenidate and amphetamine; Yang et al. (2013) suggest that GAT1 KO mouse is a new animal model for ADHD studying and GAT1 may be a new target to treat ADHD. Schmitt and Hiemke (2002) note that GABA is cleaved from the synaptic cleft by uptake (see Hu and Quick, 2008), via specific transporters and inhibition of such transporters increases the effectiveness of physiologically released GABA. Increased GABAergic neurotransmission has an impact on learning and memory. Indeed, tiagabine, a GABA-transporter inhibitor, impaired learning (Morris water-maze) and retrieval (only at the probe trial; Schmitt and Hiemke, 2002). But, Sałat et al. (2015) note that tiagabine slightly decreased memory but did not augment that induced by scopolamine. According with Shi et al. (2012), homozygous GAT1^(−/−)^ mice exhibit impaired hippocampus-dependent learning and memory; and they evaluated the impact of endogenous reduced GABA reuptake on cognitive behaviors. Learning and memory of heterozygous GAT1^(+/−)^ mice was determined in passive avoidance and Morris water maze; showing that GAT1(+/−) mice displayed increased learning and memory, decreased anxiety-like behaviors, and highest synaptic plasticity relative to wild-type and homozygous GAT1^(−/−)^ mice; and authors conclude that a moderate reduction in GAT1 activity is associated to learning and memory facilitation (Shi et al., 2012); which is consistent, in part, with GAT1 reduced and increased expression in autoshaping amnesia, forgetting and improved memory as well as anti-amnesic and anti-forgetting effects (see Table 10). In addition, Pang et al. (2011) testing the GABAergic immunotoxin; GAT1-saporin (GAT1-SAP), report no alterations in spatial reference memory. But GAT1-SAP impaired the platform location in a delayed match to position test (changing daily the platform location). In the active avoidance task, intraseptal GAT1-SAP impaired extinction but not acquisition (Pang et al., 2011). In contrast, GAT1-Saporin into the medial septum/vertical limb of the diagonal band (MS/VDB) spared mnemonic function and use of environmental cues; however, self-movement cue processing was compromised (Köppen et al., 2013).

**Table 10:** **GABA transporter GAT1**.

**Function/dysfunction**	**Findings**	**References**
GAT1 KO mice and ADHD	Hyperactive behavior and memory dysfunctions in the MWM, also have low levels of attention and increased impulsivity	Yang et al., 2013
GABA-transporter inhibitor	Tiagabine, in the MWM, compared to saline treated rats, impaired learning during the acquisition trials. And retrieval only at the probe trial	Schmitt and Hiemke, 2002
GAT1^(−/−)^ KO mice	Impaired hippocampus-dependent learning and memory (MWM, PA)	Shi et al., 2012
GABAergic immunotoxin: GAT1-saporin (GAT1-SAP)	Intraseptal impaired a delayed match to position task and extinction of avoidance without altering acquisition of WMWM, active avoidance acquisition or open field behavior. Also, animals were slower to update changes to previous contingencies	Pang et al., 2011; but see Köppen et al., 2013
GAT1(+/−) mice	Increased learning and memory, decreased anxiety-like behaviors, and highest synaptic plasticity compared with wild-type and homozygous GAT1(−/−) mice	Shi et al., 2012

*ADHD, attention deficit and hyperactivity disorder; KO, knock out; MWM, Morris water maze; PA, passive avoidance*.

### EAAT1

According with Chen et al. (2011), an imbalance of neurotransmitters (e.g., glutamate, acetylcholine, dopamine, and serotonin) has been proposed as the neurobiological basis of behavioral symptoms of AD, hence they are hypothesizing that altered reuptake of neurotransmitters by vesicular glutamate transporters (VGLUTs), excitatory amino acid transporters (EAATs), the vesicular acetylcholine transporter (VAChT), SERT or DAT. Examining protein and mRNA levels of these transporters in post-mortem prefrontal cortex from patients and matched non-AD controls, Chen et al. (2011) found that protein and mRNA levels of VGLUTs, EAAT1-3, VAChT, and SERT are reduced in AD, without changing DAT (Table 11). Chen et al. (2011) conclude that the reduced VAChT expression could contribute to cholinergic deficit in AD and altered neurotransmitter transporters could contribute to the pathophysiology of AD; which are potential targets for therapy (Chen et al., 2011).

**Table 11:** **Glutamate transporter 1 and markers**.

**Function/dysfunction**	**Findings**	**References**
Glutamate and AD	Reduced mRNA levels of VGLUTs, EAAT1-3 proteins	Chen et al., 2011
MDMA	Improved expression of GluR2 receptor, mGluR1, mGluR5, NR1, NR2A, NR2B and EAAT1, EAAT2-2 transporters. Increased mRNA levels of GluR3, NR2A and NR2B in caudate putamen. GluRl is reduced in the hippocampus, in hypothalamus increases expression of GluRl, GluR3, and mGluR3 mGluR	Kindlundh-Högberg et al., 2008, 2010
Mild stress model induced	Mice heterozygous (+/− VGLUT1) VGLUT1 decrease expression relative to wild mice: dysfunctions in recognition memory (recognition new object); anhedonia (sucrose intake), hopelessness (forced swimming), anxiety (elevated plus maze)	Garcia-Garcia et al., 2009
Glutamate transporter 1 and training	The hippocampal levels of GLT-1 complex are parallel to training in the memory multiple T-maze test	Heo et al., 2012

*AD, Alzheimer's disease; VGLUTs, Vesicular glutamate transporters; MDMA, methylenedioxymethamphetamine*.

Likewise, Kindlundh-Högberg et al. (2010) investigated the effect of intermittent 3,4-methylenedioxy-metamphetamine (MDMA; ecstasy) administration upon gene-transcript expression of the glutamate transporters (EAAT1, EAAT2-1, EAAT2-2), glutamate receptor subunits of AMPA (GluR1, GluR2, GluR3), glutamate receptor subunits of NMDA (NR1, NR2A, and NR2B), and metabotropic glutamate receptors (mGluR1, mGluR2, mGluR3, mGluR5); showing increased cortical expression of GluR2, mGluR1, mGluR5, NR1, NR2A, NR2B, EAAT1, and EAAT2-2 (Kindlundh-Högberg et al., 2010). In the caudate putamen, mRNA levels of GluR3, NR2A, and NR2B receptor subunits are increased; in contrast, GluR1 is reduced in the hippocampus but in the hypothalamus GluR1, GluR3, mGluR1, and mGluR3 expression is increased (Kindlundh-Högberg et al., 2010; see also Carmona et al., 2009); concluding that repeated MDMA administration is associated with changes in gene-transcript expressions of glutamatergic NMDA and AMPA receptor subunits, metabotropic receptors and transporters in brain areas mediating learning and memory (Kindlundh-Högberg et al., 2010). In addition, decreased expression of vesicular glutamate transporter 1 (VGLUT1+/−) respect to wild-type (WT) mice occur with chronic mild stress (CMS)-induced, affecting several functions and impairing recognition memory. In addition, Heo et al. (2012) detect hippocampal glutamate transporter 1 (GLT-1) complex expression during training and memory in the Multiple T-maze.

### SERT

Reichel et al. (2012) report that control rats spent more time interacting with the objects in the changed locations. In contrast, contingent or non-contingent methamphetamine (meth) disrupted object-in-place (OIP) task performance as seen by similar amounts of time spent with all objects, regardless of location. While only acute meth binge produced signs of neurotoxicity, both meth regimens decreased SERT in the perirhinal cortex and hippocampus. Only meth self-administration resulted in a selective decrease in NET. Meth-induced changes in SERT function in the OIP circuitry may underlie memory deficits independently of overt neurotoxic effects (Reichel et al., 2012). It should be noted that SERT is reduced in AD (Chen et al., 2011; Claeysen et al., 2015).

Parrott (2013) highlights that decreased SERT (hippocampus, parietal cortex, and prefrontal cortex expression) in abstinent Ecstasy/MDMA users is associated to dysfunctional declarative and prospective memory. Even the children of mothers who take Ecstasy/MDMA during pregnancy have psychomotor impairments (Parrott, 2013). In addition, Thomasius et al. (2006) report reduced SERT expression, which might be a transient effect of heavy ecstasy use, since it partially recovered as the users reduced their MDMA use; though this parameter may not necessarily be a valid indicator of the number or integrity of serotonergic neurons. Importantly, ex-ecstasy users' verbal memory show no sign of improvement even after over 2.5 years of abstinence and thus may represent persistent functional consequences of MDMA neurotoxicity; alternative causes such as pre-existing group differences cannot be excluded (Thomasius et al., 2006). In addition, AD and drugs of abuse like d-methamphetamine (METH) or MDMA have been associated to decrements in the SERT expression and memory deficits; thus supporting the notion that the SERT plays a key role in both normal and pathological states (e.g., Line et al., 2014). Particularly, the s allele of the polymorphic regulatory region of the SERT or 5-HTT gene promoter is associated with reduced 5-HTT expression and vulnerability to psychiatric disorders, including anxiety and depression. Moreover, the l allele increases 5-HTT expression and is generally considered protective (Line et al., 2014). However, Line et al. (2014) suggest that 5-HTT over-expression results in a reduced sensitivity to both positive and negative reinforcers, and produces some maladaptive effects, supporting recent suggestions that l allele homozygosity may be a potential risk factor for disabling psychiatric traits (Line et al., 2014). In contrast, increased 5-HTT expression reduces negative cognitive bias for stimuli with uncertain outcomes (McHugh et al., 2015). And Brigman et al. (2010) report that fluoxetine-treated C57BL/6J mice made fewer errors than controls during the early phase of learning reversal when perseverative behavior is relatively high and 5-HTT null mice made fewer errors than controls in completing the reversal task (Table 12). And these authors suggest that inactivating 5-HTT improves reversal learning, which is relevant for the pathophysiology and treatment of neuropsychiatric disorders characterized by executive dysfunction (Brigman et al., 2010) and possibly post-traumatic stress disorder.

**Table 12:** **Serotonin transporter SERT**.

**Function/dysfunction**	**Findings**	**References**
Methamphetamine	Memory deficits and decreased SERT function in perirhinal cortex and hippocampus	Reichel et al., 2012
MDMA (ecstasy)	During abstinence memory deficits and decreased SERT in hippocampus, parietal cortex and prefrontal cortex	Parrott, 2013
MDMA ex-users	Verbal memory dysfunction even after 2.5 years of abstinence	Thomasius et al., 2006
AD	Decreased SERT	Chen et al., 2011
5-HT re-uptake inhibition	In healthy individuals and aged transgenic AD mice model (APP/PS1 plaque-bearing mice), citalopram decreased Aβ in brain interstitial fluid in a dose-dependent manner	Sheline et al., 2014a,b
5-HT uptake inhibitor or SERT^(−/−)^ KO mice	Pharmacological or genetic inactivation of the serotonin transporter improves reversal learning in mice	Brigman et al., 2010
Expression	Overexpression of SERT reduces sensitivity to both positive and negative reinforcers evidence in CER and the T-maze; this overexpression is maladaptive effects, suggesting that the homozygous allele/can cause disabling psychiatric features	Line et al., 2014
Expression	Increased 5-HTT expression reduces negative cognitive bias for stimuli with uncertain outcomes	McHugh et al., 2015

*AD, Alzheimer's disease; MDMA, methylenedioxymethamphetamine; CER, conditioned emotional response*.

Certainly, SERT is providing useful information as neural marker and therapeutic target. For instance, Wallace et al. (2014) report that vortioxetine, a novel, multimodal-acting antidepressant, is a 5-HT_3_, 5-HT_7_, and 5-HT_1D_ receptor antagonist, a 5-HT_1B_ receptor partial agonist, a 5-HT_1A_ receptor agonist, and inhibits the 5-HT transporter. This drug changes the expression of multiple genes involved in neuronal plasticity by antidepressant treatment, which is associated with improved cognitive function and a reduction in depression-like behavior in middle-aged mice (Li et al., 2015c).

Hence, the SERT expression seems to be a reliable neural marker related to memory mechanisms, its alterations and potential treatment (Meneses, 2013). Resulting crucial determining the pharmacological, neural and molecular mechanisms associated to these changes and therapeutic targets. For instance, Sheline et al. (2014a) report that serotonin signaling suppresses generation of amyloid-β (Aβ) *in-vitro* and in animal models of AD and healthy individuals. In fact, in an aged transgenic AD mouse model the antidepressant citalopram (5-HT uptake inhibitor) in dose-dependent manner decreased Aβ in cerebrospinal fluid, suggesting AD prevention trials (Sheline et al., 2014a,b).

### DAT

According with Mereu et al. (2013), modafinil (MOD) and its R-enantiomer (R-MOD) are used for narcolepsy and sleep disorders; and also employed, off-label used as cognitive enhancers in individuals with mental disorders, including substance abusers that demonstrate impaired cognitive function. Their mechanisms of action include inhibition of dopamine (DA) reuptake via the DAT in diverse brain areas (Mereu et al., 2013; Table 13). Importantly, memantine (MEM), a dual antagonist of NMDA and alpha7 receptors, is neuroprotector against MDMA in rats, and it also prevents MDMA effect on SERT functionality and METH effect on DAT (Escubedo et al., 2009). Moreover, Söderqvist et al. (2012) have noted that dopamine plays an important role not only in dysfunctional working memory (WM) but also for improving it, including variation in DAT1, improving WM and fluid intelligence in preschool-age children following cognitive training; concluding with the role of dopamine in determining cognitive plasticity (Söderqvist et al., 2012). Ruocco et al. (2014) report that 5-HT_7_ receptor stimulation (low doses) was associated to among other findings reduced horizontal activity and (at higher dose) increased selective spatial attention, the DAT levels were decreased (low dose), and modulated expression of NMDA receptors.

**Table 13:** **Dopamine transporter DAT**.

**Function/dysfuntion**	**Findings**	**References**
Cognition	Variations in DAT1 influence the improvement of working memory in preschool children after cognitive training	Söderqvist et al., 2012
Dopamine inhibition	Modafinil is dopamine inhibitor can improve cognition in people with mental disorders who use substances abuse	Mereu et al., 2013
Modulated DAT expression in animal model of ADHD	Improved selective spatial attention	Ruocco et al., 2014

*ADHD, attention deficit and hyperactivity disorder*.

It should be noted that, before the perspective of the absence of effective treatments for dysfunctional memory and regardless the mechanisms; environmental interventions and exercise (physical and cognitive) seem offer feasible approaches (e.g., Mora, 2013; Mo et al., 2015).

## Conclusions

Of course if the above findings are replicated over time, across countries and in different experimental settings, they might provide insights about serotonin and other neurotransmission systems presenting convergent changes in diverse neural markers and signaling; thus, allowing the study of different brain functions and dysfunctions, including memory. Hence, diverse approaches might support the translatability of using neural markers and cerebral functions and dysfunctions (e.g., memory formation, AD, MCI). Likewise, hypothesis and theories (e.g., Borroto-Escuela et al., 2015) might provide appropriate limits and perspectives of the diversity of evidence. Certainly, at least, 5-HT_1A_, 5-HT_4_, 5-HT_5_, 5-HT_6_, and 5-HT_7_ receptors as well as SERT seem to be useful as neural markers and therapeutic targets.

### Conflict of interest statement

The author declares that the research was conducted in the absence of any commercial or financial relationships that could be construed as a potential conflict of interest.
